# Sequence-Dependent T:G Base Pair Opening in DNA Double Helix Bound by Cren7, a Chromatin Protein Conserved among Crenarchaea

**DOI:** 10.1371/journal.pone.0163361

**Published:** 2016-09-29

**Authors:** Lei Tian, Zhenfeng Zhang, Hanqian Wang, Mohan Zhao, Yuhui Dong, Yong Gong

**Affiliations:** 1 Department of general surgery, Navy General Hospital, Beijing 100048, China; 2 State Key Laboratory of Microbial Resources, Institute of Microbiology, Chinese Academy of Sciences, Beijing 100101, China; 3 Beijing Synchrotron Radiation Facility, Institute of High Energy Physics, Chinese Academy of Sciences, Beijing 100049, China; Florida International University, UNITED STATES

## Abstract

T:G base pair arising from spontaneous deamination of 5mC or polymerase errors is a great challenge for DNA repair of hyperthermophilic archaea, especially Crenarchaea. Most strains in this phylum lack the protein homologues responsible for the recognition of the mismatch in the DNA repair pathways. To investigate whether Cren7, a highly conserved chromatin protein in Crenarchaea, serves a role in the repair of T:G mispairs, the crystal structures of Cren7-G**T**AATT**G**C and Cren7-G**T**GATC**G**C complexes were solved at 2.0 Å and 2.1 Å. In our structures, binding of Cren7 to the AT-rich DNA duplex (G**T**AATT**G**C) induces opening of T2:G15 but not T10:G7 base pair. By contrast, both T:G mispairs in the GC-rich DNA duplex (G**T**GATC**G**C) retain the classic wobble type. Structural analysis also showed DNA helical changes of G**T**AATT**G**C, especially in the steps around the open T:G base pair, as compared to G**T**GATC**G**C or the matched DNAs. Surface plasmon resonance assays revealed a 4-fold lower binding affinity of Cren7 for G**T**AATT**G**C than that for G**T**GATC**G**C, which was dominantly contributed by the decrease of association rate. These results suggested that binding of Cren7 to DNA leads to T:G mispair opening in a sequence dependent manner, and therefore propose the potential roles of Cren7 in DNA repair.

## Introduction

Thymine-guanine (T:G) and uracil-guanine (U:G) base pairs are generated in DNA either by the spontaneous hydrolytic deamination of 5-methyl cytosine (5mC) and cytosine (C) respectively [1] or by misincorporation during DNA replication[2]. Deamination of pyrimidines occurs approximately 200–300 events per cell per day, an about 50-fold higher rate than that for purines[3]. These mismatches cause C to T transition mutations in 50% of the progeny DNA if not repaired upon DNA replication[4]. There seems to be no problems in the case of the U:G mispair, since uracil, which is not a natural DNA base, is efficiently removed in a base-excision repair pathway initiated by uracil DNA-glycosylase (UDG)[5,6]. T:G mispair presents a greater challenge, however, as the thymine arising from deamination of 5mC is indistinguishable from other thymines in the genomic DNA.

Organisms have evolved DNA repair pathways to process T:G mispairs, including mismatch repair (MMR), base-excision repair (BER) and nucleotide excision repair (NER) pathways. Crenarchaea, a major linage of hyperthermophilic archaea, lacks the critical components of the highly conserved NER and MMR pathways[7,8]. Despite the presence of the structure-specific nucleases of eukaryotic NER, the homologues of damage-recognition proteins are missing. Similarly, Crenarchaeota does not encode orthologues of MMR-specific proteins, MutS and MutL, which initiate the MMR pathway[9]. For BER pathway, the thymine DNA-glycosylase (TDG) plays a central role. The enzymes demonstrating TDG activities, however, were only identified from *Pyrobaculum aerophilum* [10] and *Aeropyrum pernix* [11] among Crenarchaea.

Chromatin proteins have been reported to be directly involved in DNA repair. In bacteria, histone-like protein HU from *Escherichia coli* was found to serve roles in repair of abasic sites and the closely opposed lesions[12,13]. Eukaryotic histones also participate in various DNA repair pathways, including MMR and DNA double-strand break repair, through regulation of their methylation and acetylation[14,15]. Crenarchaea, one of the two major phyla of cultured archaea, possesses a number of small and basic chromatin proteins, including Sul7d, CC1 and Cren7[16, 17]. And Cren7 is the unique one conserved at the kingdom level. In *Sulfolobus solfataricus* P2, a model strain of Crenarchaeota, Cren7 is synthesized in abundance, constituting ~1% of the total cellular proteins [1]. This protein preferentially binds dsDNA over ssDNA and changes the DNA geometry *in vitro* [1]. Moreover, the protein is found to be methylated dynamically at multiple sites *in vivo*[18].

Here we report the crystal structures of Cren7 in complex with two DNA sequences containing T:G mispairs. In crystals, binding of Cren7 to the AT-rich DNA duplex (G**T**AATT**G**C) induced opening of T2:G15 but not T10:G7 base pair. By contrast, both T:G mispairs in the GC-rich DNA duplex (G**T**GATC**G**C) retained classic wobble type. SPR assays showed a 4-fold lower binding affinity of Cren7 for G**T**AATT**G**C than that for G**T**GATC**G**C. These results suggested that binding of Cren7 to DNA leads to T:G mispair opening in a sequence dependent manner.

## Materials and Methods

### Protein expression and purification

The recombinant Cren7 protein was expressed and purified by using a modification of the method described previously [1]. Briefly, cell lysates were prepared by sonication in 20 mM Tris-Cl, pH 6.8, and 1 mM EDTA (buffer A). After centrifugation, the supernatant was heat-treated at 80°C for 20 min and centrifuged again. The sample was applied to a HiTrap SP Sepharose XL column (5 ml, GE) equilibrated in buffer A, and eluted successively with 200 and 500 mM KCl. Proteins eluted in 500 mM KCl were dialyzed to buffer A, concentrated to above 20 mg/ml and stored at -80°C.

### Preparation of oligonucleotides

All the oligonucleotides used in the present work were commercially synthesized at Sangon BioTech (Shanghai, China). The octamers, 5’-G**T**AATT**G**C, 5’-G**T**GATC**G**C and 5’-GCGATCGC, were dissolved in 20 mM Tris-Cl, pH 6.8, 100 mM NaCl and 1 mM EDTA to a final concentration of 10 mM oligonuleotide. Duplex DNA fragments for crystallization were prepared by allowing each octamer to self-anneal in a process involving heating at 95°C for 5 minutes in water bath and subsequent cooling to room temperature in two hours. For SPR analysis, DNA duplexes with a 5’-(dA)_8_ overhang were prepared by annealing each octamer to the 5’-biotin labeled oligomer containing its sequence (5’-biotin-AAAAAAAAG**T**AATT**G**C, 5’-biotin-AAAAAAAAG**T**GATC**G**C and 5’-biotin-AAAAAAAAGCGATCGC) in a molar ratio of 1.5:1.

### Crystallization, data collection and structure determination

Crystals of Cren7 in complex with d(G**T**AATT**G**C)_2_ or d(G**T**GATC**G**C)_2_ duplexes were grown at 20°C using sitting-drop vapor diffusion. The protein and the duplex DNA were mixed at a molar ratio of 1:1.5 to a final concentration of 1.5 mM for the protein. The sample (1 μl) was mixed with the reservoir solution (1 μl) and equilibrated with 30% PEG1500 at 20°C. The crystals were mounted on nylon loops and immediately frozen in liquid nitrogen. The data were collected to 2.0 Å and 2.1 Å at the BL17U beamline of the Shanghai Synchrotron Radiation Facility (Cren7-G**T**GATC**G**C) and in-house *Cu Kα* X-rays generated by a Rigaku MicroMax-007 rotating-anode X-ray source (Cren7-G**T**AATT**G**C). Two sets of data were integrated and scaled with HKL2000 [19].

The structures of Cren7-d(G**T**AATT**G**C)_2_ and Cren7-d(G**T**GATC**G**C)_2_ were determined by molecular replacement using the program Phaser[20] from the CCP4 program suite[21] using the crystal structure of Cren7-d(GTGATCAC)_2_ (PDB code: 3LWH) as the initial model. The software packages Refmac [22] and Coot [23] were used to complete the model. MolProbity was used to validate the structure [24]. The Ramachandran plots showed that 98.2% of the residues were in the most favorable region, and that no residues were in the disallowed region. Statistics for data collection and refinement are given in Table 1. DNA conformations were analyzed using the program X3DNA [25]. All images were prepared using Pymol (http://www.pymol.org).

**Table 1:** Data collection and refinement statistics.

Molecule	Cren7-GTAATTGC	Cren7-GTGATCGC
**Data collection**		
Space group	C222_1_	C222_1_
Cell dimensions		
*a*, *b*, *c* (Å)	51.8, 52.9, 90.7	77.7, 77.7, 104.3
*α*, *β*, *γ* (°)	90.0, 90.0, 90.0	90.0, 90.0, 90.0
Resolution (Å)	50–2.0 (2.07–2.00)^a^	50–2.1 (2.18–2.10)
*R*_merge_	0.052 (0.140)	0.049 (0.324)
*I*/σ(*I)*	42.9 (16.4)	37.6 (3.8)
Completeness (%)	96.0 (89.8)	99.9 (100)
Redundancy	6.5	8.1
**Refinement**		
Resolution (Å)	15–2.0	40–2.1
No. reflections	8,354	17,794
*R*_work/_*R*_free_	0.198/0.230	0.196/0.210
No.of atoms		
Protein	451	926
DNA	324	648
Water	97	126
B-factors	42	44.7
R.m.s deviations		
Bond lengths (Å)	0.008	0.009
Bond angles (°)	1.06	1.21
Ramachandran favoured (%)	98.2	98.2

^a^ The values in parenthesis refer to the highest resolution shell.

### Surface plasmon resonance assays (SPR)

SPR experiments were carried out at 25°C using the BIAcore 3000 instrument (BIAcore AB, Uppsala, Sweden). The running buffer contained 50 mM Tris-Cl, pH 7.5, 100 mM NaCl, 1 mM EDTA and 0.005% (v/v) Tween 20. The DNA duplexes with a 5’-(dA)_8_ overhang at one of the strands (described in section 2.2) were immobilized on the SA sensor chip to 90–100 response units (RU). The presence of the overhang was supposed to avoid the steric hindrance during Cren7 binding to DNA on the surface of the chip, without affecting the results of SPR experiments due to the low affinity of Cren7 to single-stranded DNA. A blank flow cell was used as the reference to correct for instrumental and concentration effects. Cren7 at a concentration within a range spanning the *K*_D_ value for the interaction of the protein with the DNA was injected over the DNA surface for 2 min at a flow rate of 30 μl/min. After the dissociation phase (2–4 min), bound protein was removed by a 30-sec wash with 0.01% SDS, followed by a 60-sec buffer injection. For each experiment, the measurement was repeated once at the protein concentration of 1.25 μM. Equilibrium and kinetic constants were calculated by a global fit to 1:1 Langmuir binding model (BIA evaluation 4.1 software).

## Results

### Overall structures of Cren7-DNA complexes

To investigate the structural details of the DNA containing T:G base pairs, the crystal structures of Cren7-d(G**T**AATT**G**C)_2_ (PDB code: 5K07) and Cren7-d(G**T**GATC**G**C)_2_ (PDB code: 5K17) have been determined and refined at 2.0Å and 2.1Å, respectively. A summary of data collection and final refinement statistics are listed in Table 1. The two complexes have different crystal packing patterns. In the crystal of Cren7-G**T**AATT**G**C, one protein-DNA complex is found in the asymmetric unit. Meanwhile, two complexes are in the asymmetric unit of Cren7-G**T**GATC**G**C, which are related by a perfect non-crystallographic 2-fold symmetry axis with a root-mean-square deviation (RMSD) value of 0.018 Å between them.

**Fig 1:**
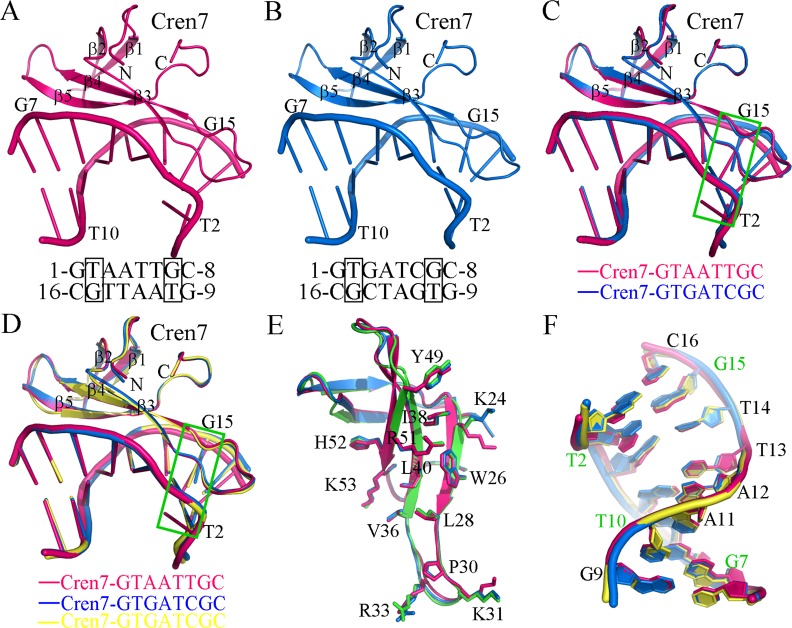
Overall structures of Cren7 in complex with DNA sequences containing T·G base pairs. (A) Ribbon diagram of the Cren7-GTAATTGC complex. (B) Ribbon diagram of Cren7-G**T**GATC**G**C complex. (C) Superposition of the two Cren7-DNA complexes, fitted by C-α atoms of protein. The T2·G15 base pairs in the two complexes are highlighted by a green box. (C) Superposition of the structures of the Cren7-GTAATTGC complex (red), the Cren7-G**T**GATC**G**C complex (blue) and the Cren7-GCGATCGC complex (yellow; PDB code: 3LWI), fitted by C-α atoms of proteins. The T2·G15 base pairs in the mismatched DNA complexes are highlighted by a green box. (E) Comparison of the protein structures derived from (D). The residues involved in Cren7-DNA contacts are shown in stick. (F) Comparison of the DNA structures (GTAATTGC, red; G**T**GATC**G**C, blue; and GCGATCGC, yellow) from different Cren7-DNA complexes. The DNA duplexes were superposed by fitting all atoms in one of the DNA strands. The bases of the strand (G9-C16) are labeled and the T·G base pairs are marked in green fonts.

The two Cren7-DNA complexes share a similar overall structure with RMSD of 0.305 Å over the backbone atoms (Fig 1A–1C). A notable distinction is flipping of the base of T2 in G**T**AATT**G**C (Fig 1C). Similar to other Cren7-DNA complexes reported previously (Fig 1D), Cren7 binds to the mismatched DNA duplex in the minor groove as a monomer, covering almost all the eight base pairs. The bound Cren7 induces a sharp kink (~50°) to the major groove by the intercalation of the side chain of Leu28 into the A3A4 step of d(GTAATTGC)_2_ (S1A Fig), while a smaller kink (~48°) is observed at the G3A4 step for d(G**T**GATC**G**C)_2_ (S1B Fig). At the intercalating site, the DNA duplex is drastically widened in the minor groove by ~5.7Å with the major groove unchanged.

Structural comparison of the Cren7 molecules in different DNA complexes showed little conformational changes (Fig 1E), suggesting that the protein retains its overall conformation when binding to different DNA sequences. All DNA-interacting residues are located on the triple-stranded β-sheet and loop β3-β4 in Cren7 (Fig 1E). The side-chain conformation of these amino acid residues exhibits small variations. The patterns of protein-DNA contacts of the Cren7-G**T**GATC**G**C and Cren7-G**T**AATT**G**C complexes are almost identical to those of Cren7 in complex with matched DNAs (S2 Fig). The exception is the loop β3-β4 region of Cren7 in the G**T**AATT**G**C complex. Only three residues on the loop, A29, P30 and K31, are involved in protein-DNA contacts, whereas the hydrogen bonds between the side chain of R33 and the bases of G1 and C16 no longer exist (S2 and S3 Figs).

### T:G base pairs

**Fig 2:**
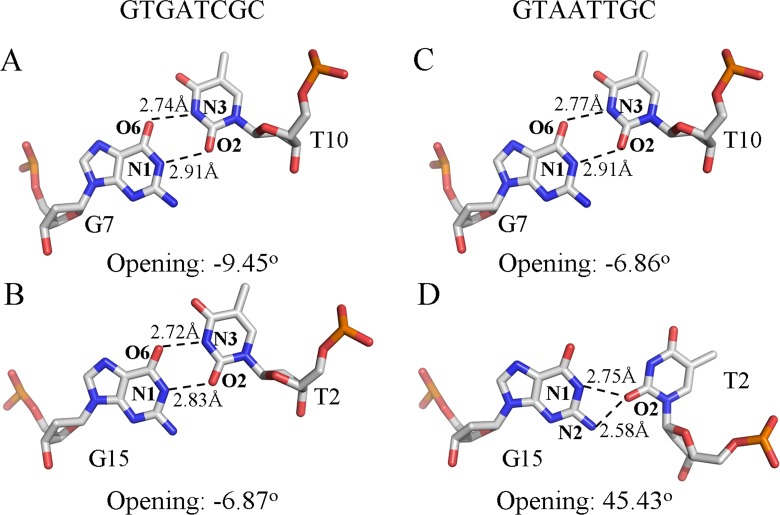
**Comparison between the classic T:G wobble base pairs (A-C) and the open T:G base pair (D).** The T:G base pairs are shown in stick view with the carbon, nitrogen, phosphorus and oxygen atoms on the coordinating chains shown in grey, blue, orange and red, respectively.

Among the most notable structural features of the Cren7-G**T**AATT**G**C complex is the flipping of the pyrimidine ring of T2 (Fig 1C). To learn more about the atomic details of the unusual T2:G15 base pairing, the structures of all the T:G mispairs in the Cren7-DNA complexes are compared (Fig 2). Both T:G base pairs in the Cren7-G**T**GATC**G**C complex form a classic wobble pair with hydrogen bonds from G-O6 to T-N3 (2.74 Å for G7:T10 and 2.72 Å for G15:T2) and from G-N1 to T-O2 (2.91 Å for G7:T10 and 2.83 Å for G15:T2) (Fig 2A and 2B). In the Cren7-G**T**AATT**G**C complex, the G7:T10 base pair is also of the wobble type with normal hydrogen bonds between G-O6 and T-N3 (2.77 Å) and between G-N1 and T-O2 (2.91 Å) (Fig 2C). By contrast, the G15:T2 base pair in this complex appears a totally different conformation with an unusual hydrogen bonding geometry instead of the wobble type (Fig 2D). The hydrogen bond from G-O6 to T-N3 has been broken, whereas the G-N1 to T-O2 hydrogen bond (2.75 Å) remains intact. Intriguingly, the hydrogen bond to T-O2 is bifurcated as an additional hydrogen bond is formed from G-N2 to this atom (2.58 Å). Although it has been reported that forming the bifurcated O2-N2 hydrogen bond is not the discriminating factor for the opening of G:U/G:T base pair [26], the existence of this hydrogen bond may contribute to the stabilization of the open state of T:G base pair.

The opening angles of the T:G base pairs are also shown in Fig 2. Notably, the opening angle of T2:G15 is ~45°, in marked contrast to the slight negative angle of other T:G base pairs. The large positive open angle of T2:G15 further confirms its open conformation in the crystal. Several other parameters, i.e. Stretch, Buckle and Propeller, of the T:G base pairs in the Cren7-G**T**AATT**G**C and Cren7-G**T**GATC**G**C complexes are compared (Table 2). The parameters of the G7:T10 base pairs in the two complexes are quite similar, indicating that the conformation of the T:G base pairs at this position are not affected by the sequence context. For the T2:G15 open base pair in Cren7-G**T**AATT**G**C, however, a largely increased value of stretch and buckle and a slightly decreased value of propeller are observed, as compared to those of the corresponding wobble base pair in Cren7-G**T**GATC**G**C.

**Table 2:** DNA local base pair parameters of the Cren7-DNA complexes.

Base pair	Stretch (Å)	Buckle (°)	Propeller (°)	Base pair	Stretch (Å)	Buckle (°)	Propeller (°)
GTAATTGC				G**T**GATC**G**C			
G:C	-0.11	-3.64	-0.11	G:C	-0.04	0.47	-2.84
T:G	1.09	15.23	-8.93	T:G	-0.63	8.95	-6.04
A:T	0.07	28.49	-13.72	G:C	-0.12	27.7	-8.20
A:T	-0.08	-16.98	1.19	A:T	-0.08	-18.8	2.26
T:A	-0.06	-4.94	-5.84	T:A	0.03	-7.95	-10.03
T:A	-0.13	2.64	-2.30	C:G	-0.12	1.49	-2.68
G:T	-0.52	-3.59	-9.52	G:T	-0.61	-4.66	-8.09
C:G	-0.07	-1.22	-5.58	C:G	-0.22	3.14	-3.40
Average	0.03	2.00	-5.60	Average	-0.22	1.29	-4.88

### DNA deformation

The mismatched DNA sequences bound by Cren7 resemble the matched ones in the global conformation except for an obvious translocation of the base of T2 in G**T**AATT**G**C (Fig 1F). The distortion of T2:G15 base pair in G**T**AATT**G**C leads to the B to A transformation of the G1pT2pA3 steps in addition to the A3pA4 step, while the later is the unique step undergoing this type of transformation in other DNA sequences bound by Cren7.

Detailed DNA conformations of the Cren7-DNA complexes are listed in Table 3. The average conformational parameters of the base pair steps in G**T**GATC**G**C resembles those in GCGATCGC bound by Cren7 [27]. It suggests that the existence of T:G mispairs has little effects on the conformation of GC-rich DNA bound by Cren7. However, the situation is quite different for G**T**AATT**G**C, an AT-rich mismatched DNA duplex, as its DNA helical parameters are significantly changed compared to those in other DNA sequences. The average roll angle of G**T**AATT**G**C (~12.1°) is much larger than that of G**T**GATC**G**C (~9.9°). In fact, the value is the largest one among all the DNA duplexes bound by Cren7 (~10.5° for GTAATTAC, ~9.9° for GCGATCGC and ~9.6° for GTGATCAC, respectively) [2,3,28], indicating that G**T**AATT**G**C is generally over curved than other DNA duplexes bound by Cren7. However, the angle of the sharp kink that occurs at the A3pA4 step in G**T**AATT**G**C (~50°) is even lower than that in GTAATTAC (~53°). The increase in DNA curvature in the Cren7-G**T**AATT**G**C complex is mainly due to the obvious positive rolls at the G1pT2pA3 steps (about 2.5° and 16.1°, respectively) since a slightly negative roll of about -0.4° and a small positive roll of 8.6° are observed at the corresponding steps in the Cren7-GTAATTAC complex.

**Table 3:** DNA helical parameters of the Cren7-DNA complexes.

Step	H-rise (Å)	Inclination (°)	Tip (°)	Shift (Å)	Tilt (°)	Roll (°)	Twist (°)
GTAATTGC							
GT/GC	3.12	3.72	-4.60	2.01	3.13	2.53	39.58
TA/TG	1.83	57.43	-11.70	-2.07	3.27	16.07	10.09
AA/TT	3.20	71.83	8.55	-0.47	-5.98	50.22	17.30
AT/AT	3.03	12.87	5.59	-0.24	-2.37	5.46	24.01
TT/AA	3.06	31.76	-8.15	-0.16	3.51	13.67	22.16
TG/TA	3.37	-2.37	1.91	-0.07	-1.34	-1.67	41.04
GC/GT	3.29	-2.54	0.67	0.59	-0.48	-1.82	41.86
Average	2.99	24.67	-1.10	-0.06	-0.04	12.07	28.02
G**T**GATC**G**C							
GT/GC	3.13	-1.31	0.00	-0.61	0.00	-0.90	40.29
TG/CG	2.81	38.48	2.59	0.36	-0.82	12.19	15.42
GA/TC	3.54	69.83	0.82	-0.84	-0.56	47.91	18.78
AT/AT	2.98	14.97	6.16	-0.38	-2.67	6.48	24.36
TC/GA	3.33	22.82	-7.18	-0.30	3.27	10.39	24.81
CG/TG	3.22	-5.66	0.67	-0.18	-0.47	-3.95	40.77
GC/GT	3.11	-4.11	1.60	0.60	-1.12	-2.89	41.10
Average	3.16	19.29	0.67	-0.19	-0.34	9.89	29.36

Binding by Cren7 also induces undertwisting of the DNA helix. Intriguingly, unlike the matched DNA sequences in complex with Cren7, the smallest twist in G**T**AATT**G**C is observed at the T2pA3 step (~10.1°) instead of the site of intercalation (A3pA4 step, ~17.3°). A similar case is found for G**T**GATC**G**C, with the values of the corresponding steps being about 15.4°and 18.8°, respectively. And, the reduction of twist at the T2pA3 step in G**T**AATT**G**C has a dominant contribution to its smallest average twist of a single step, among the DNA sequences bound by Cren7.

In addition to DNA bending and unwinding, the opening of T2:G15 base pair in G**T**AATT**G**C appears to affect some aspects of DNA conformation. The DNA helical parameters of the T2pA3 step, a step adjacent to T2:G15 base pair, in Cren7-G**T**AATT**G**C are significantly changed (Table 3). The helical-rise (H-rise) at the T2pA3 step (1.83 Å) is much smaller than that of the T2pG3 step in G**T**GATC**G**C bound by Cren7 (2.81 Å). While a largely increased inclination of ~57.4°is observed at this step in G**T**AATT**G**C, as compared to that in G**T**GATC**G**C (~38.5°), a negative tip of about -11.7° is seen here in contrast to the positive tip in the later (~2.6°).

### Kinetic analysis of the interaction of Cren7 with mismatched DNA

**Fig 3:**
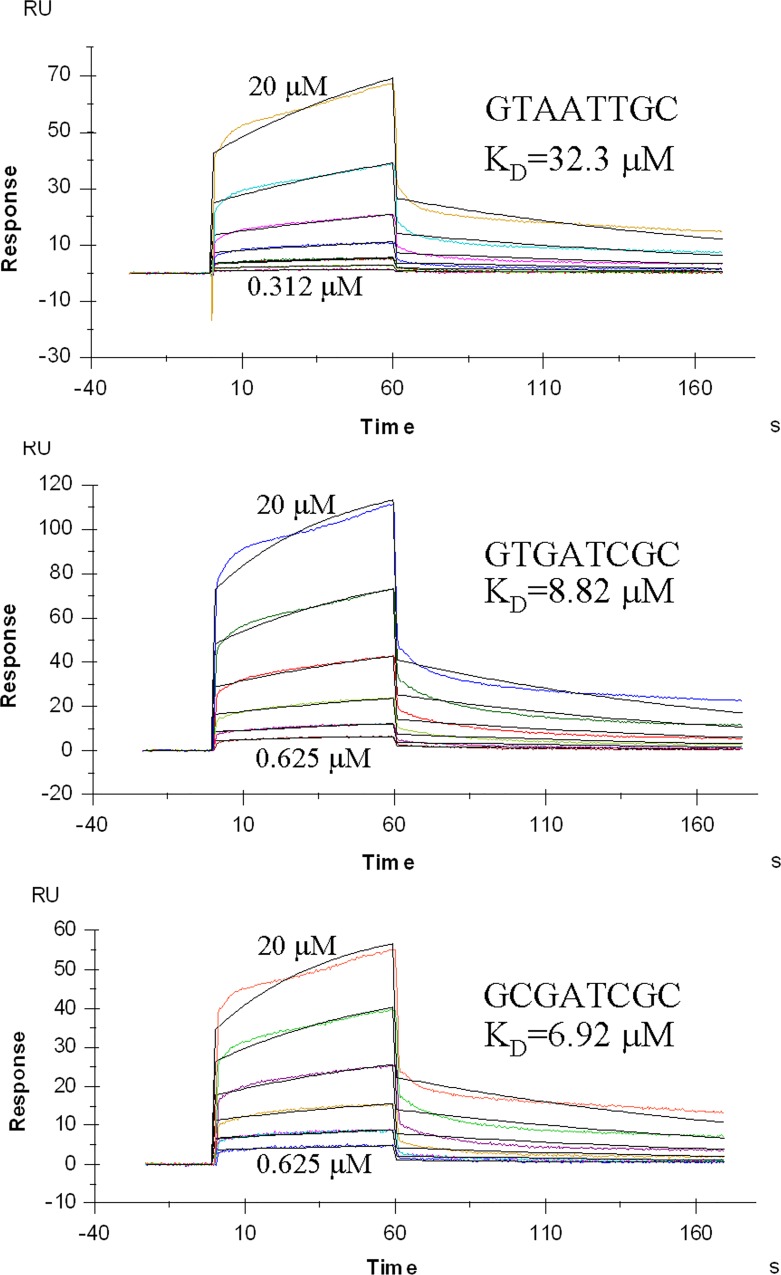
Kinetic characterization of the binding of Cren7 to mismatched or matched DNA. Cren7 was injected over immobilized DNAs. Sensorgrams are shown with the KD values labeled. RU, response units.

To learn more about the effects of T:G mispairs on the interactions between Cren7 and DNA, we analyzed the binding kinetics of Cren7 to three DNA sequences (G**T**AATT**G**C, G**T**GATC**G**C and GCGATCGC) by SPR, respectively (Fig 3). In all the experiments, a 5’-(dA)_8_ overhang of the short DNA fragments was used to avoid the steric hindrance during Cren7 binding to DNA on the surface of the chip. The presence of the single-stranded region was supposed not to affect the results of SPR experiments due to the 10-fold lower affinity of Cren7 for ssDNA [1]. Despite the low melting temperatures (10~20°C) of the DNA duplexes used in SPR assays, more than 85% of each type of the DNA fragments retained dsDNA form as revealed by polyacrylamide gel electrophoresis (data not shown). In the SPR assays, the baseline, which could represent the mass of the DNA strands immobilized on the chip surface, showed no visible decrease throughout the entire process of each experiment, suggesting that melting of the dsDNA barely occurred. In addition, the two curves derived from the repeated running cycles at the protein concentration of 1.25 μM in each experiment shown in Fig 3 were almost overlap, which further proved that the immobilized DNA duplexes remained unchanged during the measurements. Therefore, these results represented the different binding affinities of Cren7 to matched or mismatched dsDNA fragments but not the distinctions in the thermal stabilities of the DNA duplexes. Table 4 lists the kinetic parameters of Cren7 binding to different DNA sequences. Cren7 showed a 1.3-fold reduction in the binding affinity for G**T**GATC**G**C as compared to that for GCGATCGC. This was resulted from the combined effects of a slightly decreased association rate (*k*_a_) and a slightly increased dissociation rate (*k*_d_), indicating that the presence of T:G wobble base pairs had limited effects on both Cren7-DNA contacts and the stability of Cren7-DNA complex. By contrast, the binding affinity of Cren7 for G**T**AATT**G**C was 4.6-fold lower than that for the matched DNA. This reduction was dominantly led by the decrease in association rate of G**T**AATT**G**C as compared to that of G**T**GATC**G**C. These results suggest that the opening of one of the T:G mispairs largely diminishes Cren7 contacting DNA instead of reducing the stability of Cren7-DNA complex.

**Table 4:** Kinetic analysis of Cren7 binding to matched or mismatched DNA by SPR.

Sequence	Affinity K_D_ (M)	Association rate k_a_ (M^-1^s^-1^)	Dissociation rate kd (s^-1^)
GTAATTGC	3.23×10^−5^	2.25×10^2^	7.28×10^−3^
G**T**GATC**G**C	8.82×10^−6^	8.67×10^2^	7.65×10^−3^
GCGATCGC	6.92×10^−6^	9.65×102	6.69×10^−3^

## Discussion

The maintenance of genomic integrity is a crucial task for all living cells, as many environmental factors contribute to DNA damage. Of the environmental extremes accommodated by Crenarchaeota, high temperature has particular significance, as it cannot be excluded from the interior of microbial cells. High temperature directly leads to more rapid reactions such as hydrolytic deamination of nucleotide bases, which generate U:G or T:G base pairs. Despite the lack of the crucial protein homologues in MMR, NER or BER pathways, the rate of mutation in *Sulfolobus*, a model strain of Crenarchaea, is not higher than that for *Escherichia coli* [29], indicating that DNA damage is repaired efficiently.

In the present work, we resolved the crystal structures of the Cren7-G**T**AATT**G**C and Cren7-G**T**GATC**G**C complexes. In the former complex, the T2:G15 base pair shows a characteristic opening angle of ~45 °, indicating its opening state. The hydrogen bonding geometry of this base pair also resembles that of the U:G open base pair in the TGT/AUA sequence [26]. Although predictions based on pairing of the complementary bases and the stacking of pairs suggested no obvious sequence dependent base pair opening probabilities [30], Cren7 may prefer to induce the opening of T:G base pair in the AT-rich DNA sequences, as the fact that both T:G base pairs in the Cren7-G**T**GATC**G**C complex retain the classic wobble type. Therefore, Cren7 can readily induce the opening of the T:G base pairs in the genomic DNA of *Sulfolobus* cells owing to its high AT-content. Opening of the T:G base pair induces more DNA bending toward the major groove and more DNA undertwisting over other DNA duplexes bound by Cren7. The severe DNA distortions may thus affect the protein-DNA interactions, evidenced by that Cren7 shows a 4-fold lower binding affinity for G**T**AATT**G**C than for G**T**GATC**G**C. Taken together, these results suggested that opening of T:G base pair in DNA bound by Cren7 might promote the activity of some DNA repair enzymes in recognition of the mispairs and base excision of the thymine.

## Supporting Information

S1 Fig Stereoscopic view of the sites of intercalation in the Cren7-GTAATTAC (red and pink), Cren7-GTGATCGC (yellow and blue) and Cren7-GTAATTAC (PDB code: 3LWH; orange and green) complexes. The DNA octamer is kinked by ~50° at the A3pA4 step in the Cren7-G**T**AATT**G**C complex and ~48° at the G3pA4 step in the Cren7-G**T**GATC**G**C complex. The conformations of the side chains of Leu28 and Val36 in the three complexes are shown.Overall structures of Cren7 in complex with DNA sequences containing T·G base pairs.
(TIF)

S2 Fig **Schematic diagrams summarizing all of the important protein-DNA contacts in the Cren7-GTAATTGC (A) and Cren7-GTGATCGC (B) complexes.** Hydrogen bonds and hydrophobic interactions are shown with blue dashed lines and pink arrows respectively.
(TIF)

S3 Fig Stereoscopic view of the steps around the open T2:G15 base pair. The carbon, nitrogen, phosphorus and oxygen atoms on the coordinating chains of T2:G15 are shown in grey, blue, orange and red, respectively. The oxygen and nitrogen atoms involved in the hydrogen bonds are labeled. The loop β3-β4 of Cren7 is shown in cartoon with the side chains of the residues participating in the protein-DNA interactions labeled.
(TIF)
