# Supplementary figures and images for: Sequence-Dependent T:G Base Pair Opening in DNA Double Helix Bound by Cren7, a Chromatin Protein Conserved among Crenarchaea
Source: PLoS One. 2016 Sep 29;11(9):e0163361. doi: 10.1371/journal.pone.0163361 (PMC5042384; doi:10.1371/journal.pone.0163361)

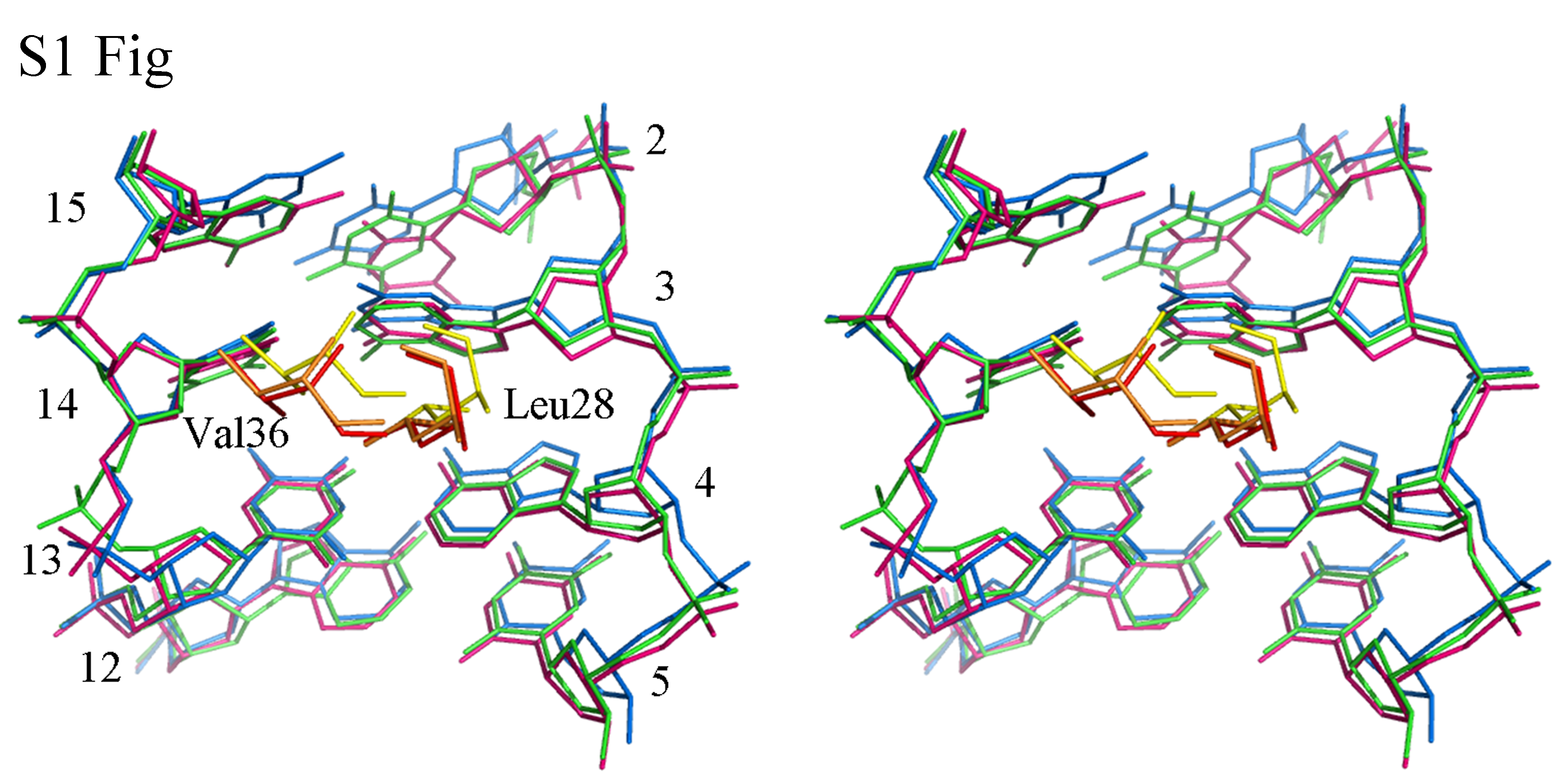

Supplement: S1 Fig — The DNA octamer is kinked by ~50° at the A3pA4 step in the Cren7-GTAATTGC complex and ~48° at the G3pA4 step in the Cren7-GTGATCGC complex. The conformations of the side chains of Leu28 and Val36 in the three complexes are shown.Overall structures of Cren7 in complex with DNA sequences containing T·G base pairs. (TIF) [file pone.0163361.s001.tif]

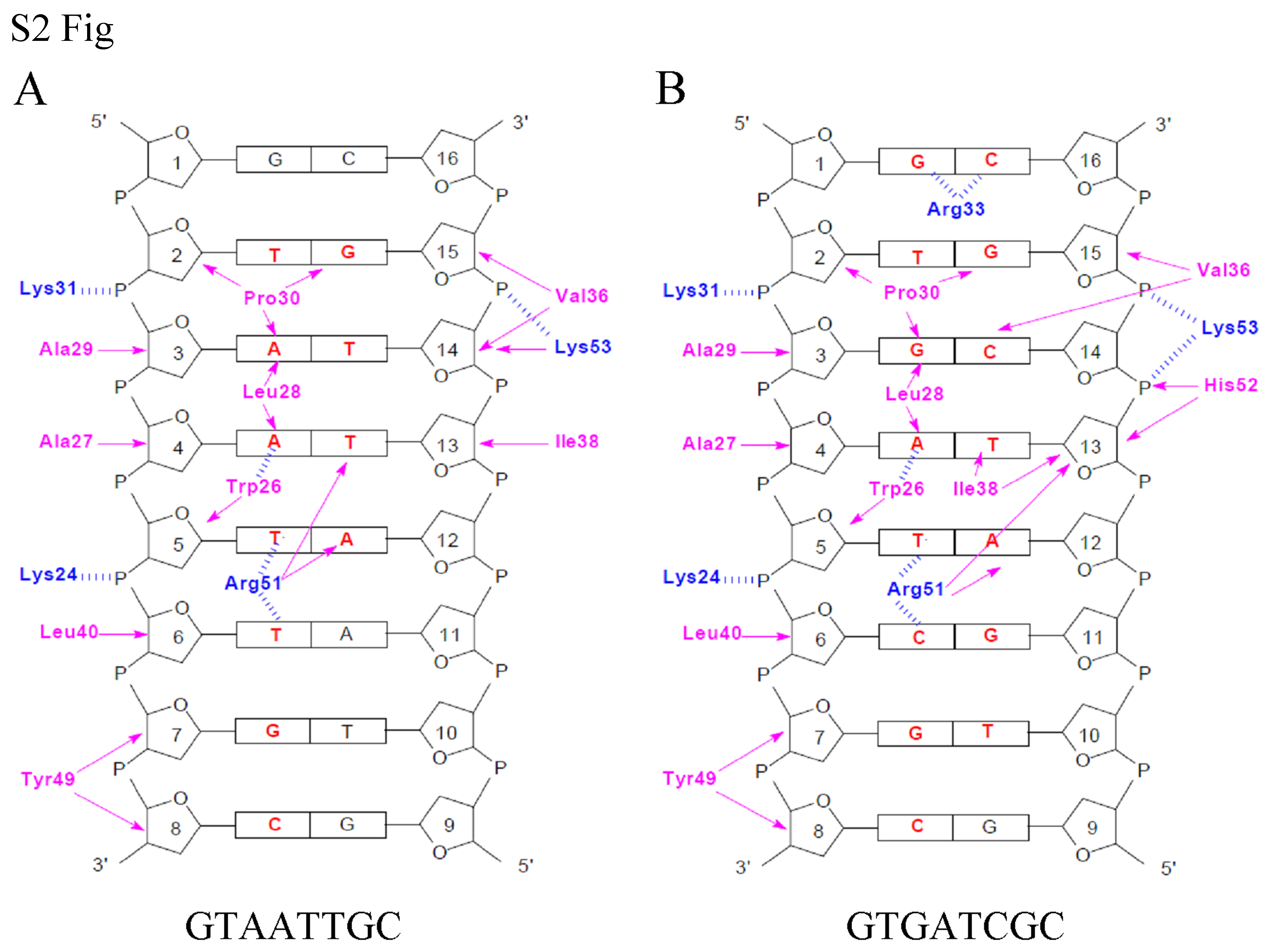

Supplement: S2 Fig — Schematic diagrams summarizing all of the important protein-DNA contacts in the Cren7-GTAATTGC (A) and Cren7-GTGATCGC (B) complexes. Hydrogen bonds and hydrophobic interactions are shown with blue dashed lines and pink arrows respectively. (TIF) [file pone.0163361.s002.tif]

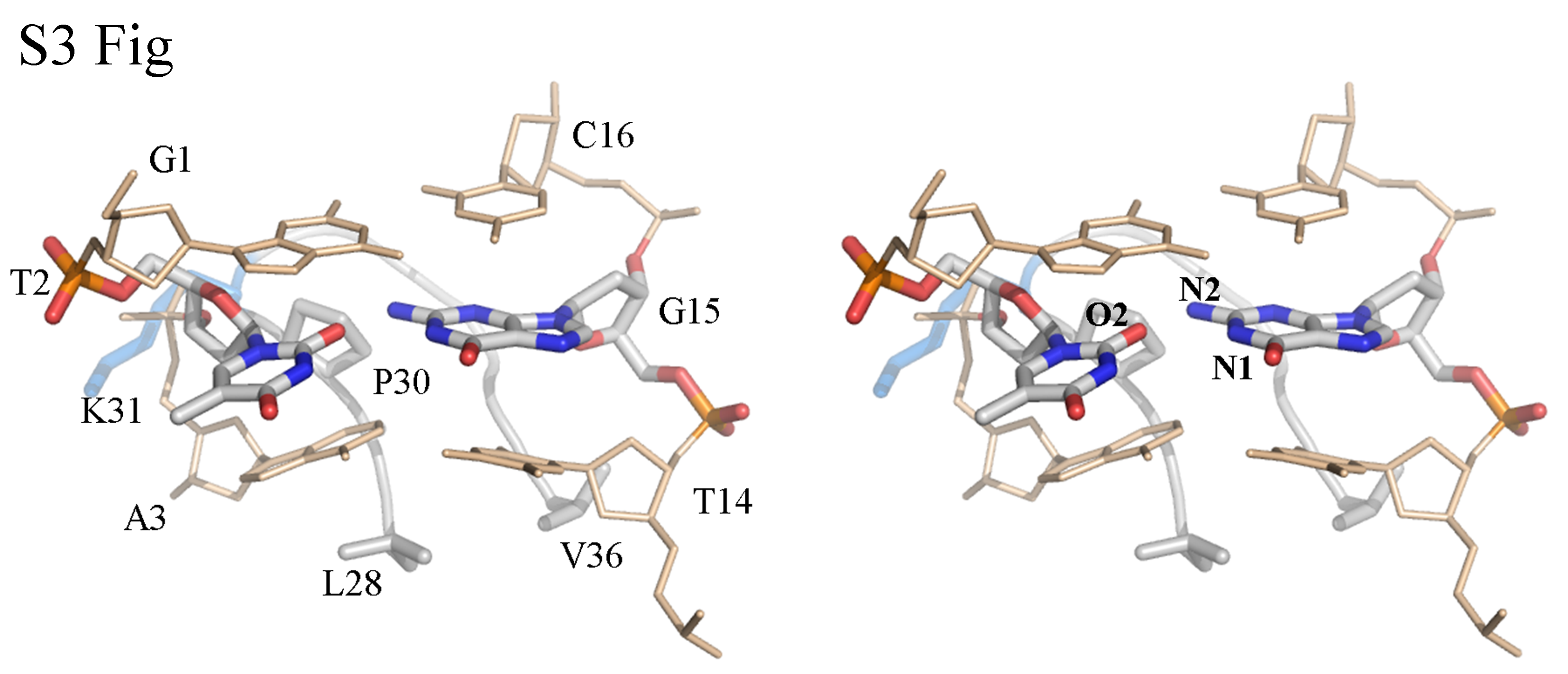

Supplement: S3 Fig — The carbon, nitrogen, phosphorus and oxygen atoms on the coordinating chains of T2:G15 are shown in grey, blue, orange and red, respectively. The oxygen and nitrogen atoms involved in the hydrogen bonds are labeled. The loop β3-β4 of Cren7 is shown in cartoon with the side chains of the residues participating in the protein-DNA interactions labeled. (TIF) [file pone.0163361.s003.tif]
